# Blood clot detection using magnetic nanoparticles

**DOI:** 10.1063/1.4977073

**Published:** 2017-02-16

**Authors:** Hafsa Khurshid, Bruce Friedman, Brent Berwin, Yipeng Shi, Dylan B. Ness, John B. Weaver

**Affiliations:** 1 Department of Radiology, Dartmouth-Hitchcock Medical Center, Lebanon New Hampshire 03756, USA; 2 Cardiology Department, Dartmouth-Hitchcock Medical Center, Lebanon New Hampshire 03756, USA; 3 Department of Microbiology and Immunology, Geisel School of Medicine, Hanover New Hampshire 03755, USA; 4 Department of Physics & Astronomy, Dartmouth College, Hanover New Hampshire 03755, USA; 5 Thayer School of Engineering, Dartmouth College, Hanover New Hampshire 03755, USA

## Abstract

Deep vein thrombosis, the development of blood clots in the peripheral veins, is a very serious, life threatening condition that is prevalent in the elderly. To deliver proper treatment that enhances the survival rate, it is very important to detect thrombi early and at the point of care. We explored the ability of magnetic particle spectroscopy (MSB) to detect thrombus via specific binding of aptamer functionalized magnetic nanoparticles with the blood clot. MSB uses the harmonics produced by nanoparticles in an alternating magnetic field to measure the rotational freedom and, therefore, the bound state of the nanoparticles. The nanoparticles’ relaxation time for Brownian rotation increases when bound [A.M. Rauwerdink and J. B. Weaver, Appl. Phys. Lett. **96**, 1 (2010)]. The relaxation time can therefore be used to characterize the nanoparticle binding to thrombin in the blood clot. For longer relaxation times, the approach to saturation is more gradual reducing the higher harmonics and the harmonic ratio. The harmonic ratios of nanoparticles conjugated with anti-thrombin aptamers (ATP) decrease significantly over time with blood clot present in the sample medium, compared with nanoparticles without ATP. Moreover, the blood clot removed from the sample medium produced a significant MSB signal, indicating the nanoparticles are immobilized on the clot. Our results show that MSB could be a very useful non-invasive, quick tool to detect blood clots at the point of care so proper treatment can be used to reduce the risks inherent in deep vein thrombosis.

## INTRODUCTION

Thrombin is a coagulation serine protease that plays a key role in thrombogenesis/hemostasis, which is the process that halts bleeding secondary to blood vessel damage.^2^ In case of an injury, thrombin triggers a reaction causing plasma-soluble fibrinogen to be transformed into non-plasma-soluble fibrin in the clot.^3^ The interaction of tissue factor with plasma factor VII activates the coagulation cascade, producing thrombin by stepwise activation of a series of proenzymes. The coagulation cascade is an essential part of hemostasis. However, the same coagulation factors can also form clots in the central veins producing deep vein thrombosis (DVT).^4^ It is a very serious condition that can cause permanent damage to the leg or a life-threating pulmonary embolism. In the United States alone, 600,000 new cases are diagnosed each year. The survival rate is excellent if the thrombus formation is detected early and treated quickly. DVT is currently diagnosed using real-time ultrasound imaging, in which ability to compress the vein along with the absence of Doppler flow signal provides indirect evidence of the clot. However, this method has some limitations in distinguishing acute from chronic DVT. Most of the other methods currently used for blood clot diagnostics also suffer from the major disadvantage of being invasive.^5,6^

In a recent report superparamagnetic iron oxide magnetic nanoparticles (MNPs) have been used for localized hyperthermia to accelerate blood clot lysis.^7^ Drozdov et al. has reported the production of biocompatible thrombolytic magnetic composite materials with non-releasing behavior and prolonged action.^8^ Magnetic particle spectroscopy (MPS) has been used as a surrogate for MPI to quantify the amount of iron on the clot^9,10^ ex vivo but has not been used to assess the binding of MNPs to the thrombus. Herein we report the development of a nanoscale biosensor system composed of MNPs conjugated with thrombin binding aptamers. We make use of the specific interactions between ATP conjugated iron oxide MNPs (MNP-strep-ata) and thrombin in the blood clot to detect blood clot.^11^ The MNPs bound to thrombus are identified by the reduction of their Brownian rotational freedom detected using magnetic spectroscopy of nanoparticle Brownian motion (MSB).

MSB is a type of MPS that is sensitive to Brownian rotation of the MNPs allowing binding and temperature effects to be quantified.^12–14^ MSB uses the harmonics of the magnetization produced when the MNPs are placed in an oscillating magnetic field.^14^ The ratio of harmonics is often used as a metric for their rotational freedom that reflects the bound state of the MNPs.^11^ The balance between the forces produced by the sinusoidal magnetic field and thermal effects makes the alignment of the magnetic moments of MNPs a sensitive indicator of binding.^17^ The higher harmonics in the MNP’s magnetization are uniquely associated with the MNPs making MSB highly sensitive as is MPI. The harmonic ratio quantifies the balance between the magnetic forces aligning the MNPs and rotational Brownian motion that tends to randomize their directions. This technique can be made sensitive to a selected molecular biomarker.^17^ When a biomarker molecule has multiple binding sites, ligands from two different MNPs can bind to the same biomarker molecule assembling the MNPs into clusters or aggregates. Such an interaction leads to an increasing effective size of the MNP cluster, resulting in increased Brownian relaxation time that can be sensed using MSB.^1,17^

Their magnetic properties can be detected in vivo and because of their biocompatibility and stability, surface functionalized iron oxide MNPs have been widely used as a powerful tool in numerous biomedical applications including biosensors and bio assays.^15^ Owing to their large surface to volume ratio, MNPs provide a relatively large number of binding sites for the aptamers and antibodies hence offering highly efficient binding. To probe, the MNPs’ binding to thrombin, the MNPs were conjugated with ATP15 and ATP29 aptamers, which bind to the two distinct epitopes on thrombin. These aptamers are oligonucleotides that fold into secondary structures and form pockets for the binding of specific ligands. Because of their high affinity and selective binding to thrombin, the ATP15 and ATP29 aptamers are very useful tools to probe the structure of thrombi.^16^ The MNPs bound to thrombus were identified by the reduction of their Brownian rotational freedom. Our data shows that MSB is a useful tool for the detection and characterization of blood clots and hence it has the potential to reduce the risks of DVT.

## EXPERIMENTAL SECTION

The spectrometer used to measure the harmonics of the MNPs magnetization has been described previously.^1,17–19^ Briefly, an alternating drive field was generated using a sinusoidal voltage, generated by an SR830 phase-locked amplifier (Zurich Instruments HF 2LI Lock in Amplifier) and amplified by an audio power amplifier (QSC PL 236) driving current through a resonant coil. A computer controlled relay bank allowed different capacitors to be used in series with the drive coil to obtain the desired resonant frequencies. The applied magnetic field induced a magnetization that was recorded by the pickup coil. The phase-lock amplifier was used to amplify and record the harmonics generated by the nanoparticles sample. A field coil was used to monitor and adjust the amplitude of the applied field, which was maintained at a constant amplitude for all frequencies.

The MNPs used in this study are 100 nm iron oxide nanoparticles synthesized by Micromod Partikeltechnologie (GmbH, Germany), product code 10-19-102 BNF starch. The particle surface is coated with streptavidin and their matrix consists of crosslinked hydroxyethyl starch. The particles measured hydrodynamic diameter size is approximately 113 nm (Malvern ZetaSizer Nano ZS, Malvern Instruments Ltd, UK) and their manufactured iron concentration is 6 mg ml. They consist of a magnetic core approximately 50 nm in diameter formed of a conglomerate of magnetite crystals coated with starch. The samples were composed of 20μl of MNPs, suspended in 200μl PBS media containing 0.05% Tween 20 as surfactant. The suspensions were placed in silicone coated 2 ml Eppendorf tubes. The surfactant and silicone coating of the tubes prevented the adherence of the MNPs to the tube walls. Animal use was approved by the Dartmouth Institutional Animal Care and Use Committee (Protocol berw.bl.2). Blood was obtained from euthanized C57BL/6 mice by cardiac puncture with a 27G needle.

Previously reported 15-mer and 29-mer^18^^,^^16^ anti-thrombin aptamer sequences with biotin modification at 5′ were used in the thrombin targeted system. Prior to use, MNPs were functionalized by conjugating the terminal biotin moieties on the aptamer to the streptavidin labeled MNPs. The details of binding has been reported previously.^13^ 10 ul of each of NP-ata15 and NP-ata29 were dissolved in 130 ul phosphate-buffered saline (PBS) and vortexed. MSB spectra were obtained at 10mT/μ_o_ using a range of frequencies from 400 to 1600 Hz. In the first step, harmonic spectra of MNP-atp were taken as control experiments. The blood clot formation was carried out at room temperature in polyvinyl plastic tubing, after venipuncture. Later, blood clots of the similar mass and size and various ages (minutes) were added to the above MNP’s sample tube, vortex and MSB spectra was obtained immediately. 10 to 60 spectra were measured. Each measurement took 1.5 minutes. In the third step, clot was removed from the sample cuvette (without taking any MNP dispersion with it), added to PBS and MSB spectra of the clot were obtained under the same experimental parameters (temp, frequency, field etc). The ratio of the 4^th^ to 2^nd^ harmonics were used as a metric of binding that is independent of the MNP concentration.

## RESULTS AND DISCUSSION

**FIG. 1.**
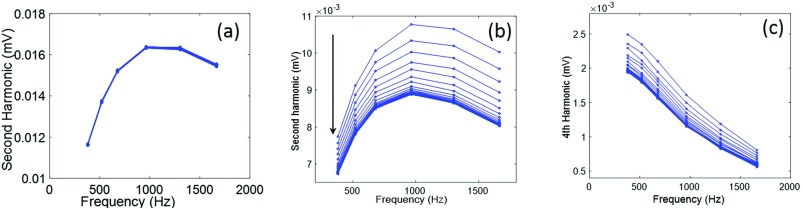
MSB measurement of 2^nd^ harmonics of NP-strep-ata samples using 5 mT/μo before (a), after adding blood clot (b), and 4^th^ harmonic (c). Increasing time is indicated using the arrow.

Figure 1 shows the MSB measurements of the second harmonic of MNP-strep-ata in PBS before (a) and after (b) adding blood clot. The reproducibility of the result is indicated by the invariance. These spectra were taken at 5mT/μ_o_ without changing the sample position. For the MNPs without any blood clot, the second harmonic does not change over the time and hence MNPs are stable over the time. MNPs with the clot produced harmonic ratios that dropped over the time and eventually reached an equilibrium state. As the binding occurs between MNPs and thrombin in the blood clot, the free rotation of MNPs is restricted and hence the relaxation time of each individual MNP increases^19^ that is reflected by the decreasing harmonics.^20^ For example, a significant drop is seen in the second harmonic, until an equilibrium state is reached that can be seen where the spectra cluster at the bottom of the plots. For short relaxation times of unbound MNPs, the magnetization saturates quickly, and a sharp corner in the magnetization is produced. For longer relaxation times, the approach to saturation is smoother and thus higher harmonics are reduced, so the ratio of any two increasing harmonics decreases.

**FIG. 2.**
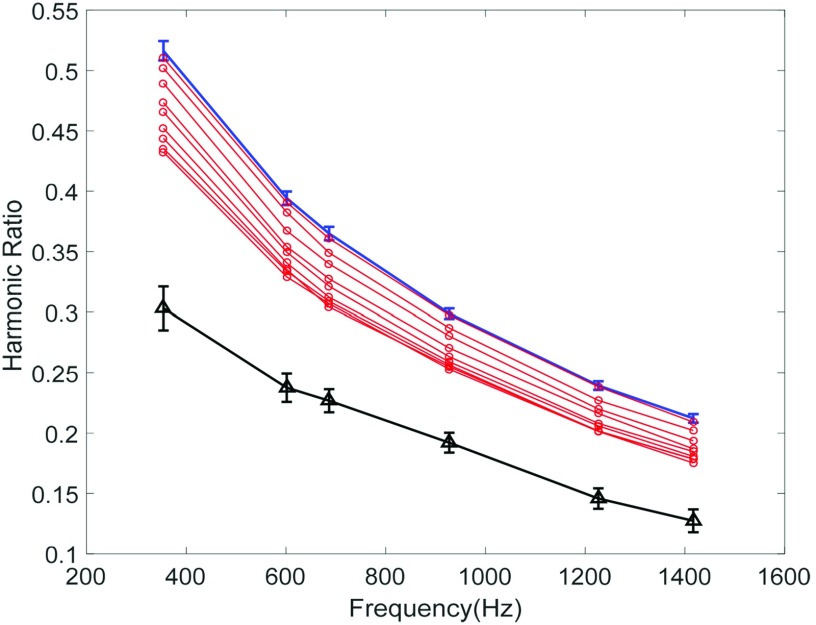
The MSB spectra of the harmonic ratios for 10 mT/μo applied fields using TAP conjugated nanoparticles along with blood clot. The blue curve marks nanoparticles before adding clot and the red curves mark signal from nanoparticles with blood clot. The signal from clot in PBS is shown in black curve with symbols △.

Figure 2 shows the MSB spectra of MNPs (NP-ata29 and NP-ata15) in PBS along with blood clot. The control measurements from MNPs conjugated with anti-thrombin aptamers is marked in blue color. Once blood clot was added to the above control sample, the harmonic ratios drop consecutively because of the binding. To confirm the MNP’s binding to blood clot, the clot was removed from MNPs’ suspension and its MSB spectrum was taken in PBS (shown in black color in Fig.2).

**FIG. 3.**
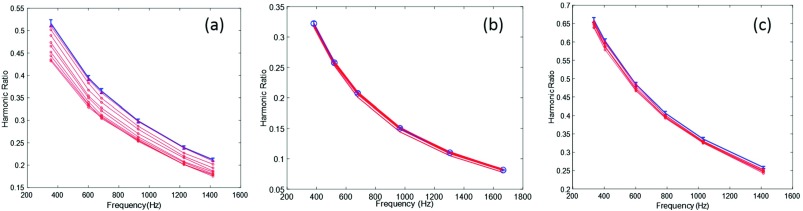
Harmonic ratios from a 1:1 mixture of NP-ata15 and NP-ata29 at 10 mT/μo along with blood clot. The clot age is 8 (a), and 120 minutes (b) after the venipuncture and blood collection. (c) is MSB harmonic ratios from the clot when nanoparticles used without ATP conjugation.

To compare the MSB signal response of the clot stiffness, the aging time of blood clots was varied. The MSB signal response upon increasing clotting time can be seen in Fig. 3. After venipuncture and blood collection, the clotting time was varied from 8 minutes to 120 minutes at room temperature. The MSB harmonic ratios drop about 20% and 2%, when an eight minutes and a 120 minutes old clot was used, respectively. After venipuncture, it takes about 10-12 minutes for the thrombin level to increase at 22 °C in polypropylene test tube.^21^^,^^6^ A significant drop in the MSB harmonic ratios indicates the MNP specific binding to thrombin within first few minutes of measurements. It is inferred that after 120 minutes of blood collection, the coagulation cascade would have been completed and not much thrombin is available for MNP binding. The 2% drop in harmonic ratios is probably due to nonspecific interactions. A control using MNPs with no targeting aptamer was used to isolate the MSB changes caused by spurious effects including pH changes from specific thrombin binding. A newly developed blood clot (10 minutes old) was used for this purpose (Fig. 3c). As it can be seen in Fig. 3c, the harmonic ratios drop is less than 3% over the time used for the previous experiments. That is comparable to the case when a mature (120 minutes older) clot was used. The small changes induced without targeting aptamers suggests that ATP conjugation plays a critical role in the MNPs binding to thrombin and MNPs do not bind to the clot without ATP conjugation.

## CONCLUSIONS

MSB is a promising technique to diagnose blood clots with the potential of functioning at the point of care. The work presented here demonstrates a new, noninvasive method for detecting blood clots using antithrombin aptamers conjugated to iron oxide MNPs. Future work will be focused on the real time measurements/detection of dynamic clot formation. Our data shows that MSB is a useful tool to detect thrombi so it is a promising technology to reduce the risks of DVT.
